# Adaptive response of neonatal sepsis-derived Group B *Streptococcus* to bilirubin

**DOI:** 10.1038/s41598-018-24811-3

**Published:** 2018-04-24

**Authors:** Richard Hansen, Sophie Gibson, Eduardo De paiva Alves, Mark Goddard, Andrew MacLaren, Anne Marie Karcher, Susan Berry, Elaina S. R. Collie-Duguid, Emad El-Omar, Mike Munro, Georgina L. Hold

**Affiliations:** 1 Department of Paediatric Gastroenterology, Royal Hospital for Children, Glasgow, G51 4TF United Kingdom; 2 Gastrointestinal Research Group, School of Medicine, Medical Sciences and Nutrition, University of Aberdeen, Foresterhill, Aberdeen, AB25 2ZD United Kingdom; 3 Neonatal Unit, Aberdeen Maternity Hospital, Foresterhill, Aberdeen, AB25 2ZL United Kingdom; 4 Centre for Genome Enabled Biology and Medicine, University of Aberdeen, 23 St Machar Drive, Aberdeen, AB24 3RY United Kingdom; 5 Department of Medical Microbiology, Aberdeen Royal Infirmary, Foresterhill, Aberdeen, AB25 2ZD United Kingdom; 6 St George and Sutherland Clinical School, University of New South Wales, Sydney, Australia

## Abstract

Hyperbilirubinemia is so common in newborns as to be termed physiological. The most common bacteria involved in early-onset neonatal sepsis are *Streptococcus agalactiae*, commonly called Group B *Streptococcus* (GBS). Whilst previous studies show bilirubin has antioxidant properties and is beneficial in endotoxic shock, little thought has been given to whether bilirubin might have antibacterial properties. In this study, we performed a transcriptomic and proteomic assessment of GBS cultured in the presence/absence of bilirubin. Our analysis revealed that increasing levels of bilirubin (>100 µmol/L) negatively correlated with GBS growth (18% reduction from 0–400 µmol/L on plate model, p < 0.001; 33% reduction from 0–100 µmol/L in liquid model, p = 0.02). Transcriptome analysis demonstrated 19 differentially expressed genes, almost exclusively up-regulated in the presence of bilirubin. Proteomic analysis identified 12 differentially expressed proteins, half over-expressed in the presence of bilirubin. Functional analysis using Gene Ontology and KEGG pathways^18^ revealed a differential expression of genes involved in transport and carbohydrate metabolism, suggesting bilirubin may impact on substrate utilisation. The data improve our understanding of the mechanisms modulating GBS survival in neonatal hyperbilirubinemia and suggest physiological jaundice may have an evolutionary role in protection against early-onset neonatal sepsis.

## Introduction

Jaundice is an extremely common finding in newborn infants after the first 24 hours, being present in between 60% of term and 80% of preterm infants^1^. Physiological jaundice is due to both an increase in the production of bilirubin, principally by haemolysis of red blood cells, and a reduction in the conjugation of bilirubin by the liver enzyme uridine diphosphate glucuronyl transferase^2^. Generally, physiological jaundice is transient and diminishes over the first week of life. In breastfed infants in particular, persistent jaundice can be found in 10% of infants at 1 month^1^.

In the majority of infants who develop jaundice it will resolve spontaneously with no ill effects; however, a rare few will develop significant complications of bilirubin encephalopathy. Acute bilirubin encephalopathy can present with a variety of clinical features including lethargy, irritability, abnormal muscle tone and, in severe cases, seizures and apnoea. Chronic bilirubin encephalopathy results in the long-term sequelae of choreoathetoid cerebral palsy and sensorineural deafness^1^. These detrimental effects of hyperbilirubinemia raise the possibility of an opposing evolutionary advantage in the first week of life, however little work has been done exploring possible benefits. Clinical tolerance of hyperbilirubinaemia is generally low because of the concern regarding encephalopathy and kernicterus, therefore bilirubin levels are not generally allowed to remain high for significant periods. Bilirubin levels of >100 µmol/l are not uncommon and they can often reach levels of >300 µmol/l untreated. The literature to date discusses only the potential benefit of bilirubin as an anti-oxidant^3,4^, however a recent paper has shown that the anti-oxidant properties appear to become pro-oxidant above around 100 µmol/l, suggesting a possible dose-dependent dichotomy of function for hyperbilirubinaemia^5^. Curiously, the creation of bilirubin from biliverdin requires a net input of energy during the reduction of biliverdin to bilirubin by biliverdin reductase^4^. Given that biliverdin would be an adequate end product of heme degradation (which indeed it is in birds, reptiles and amphibians), it seems curious that additional energy is used to convert it to potentially toxic bilirubin if it has no valuable physiological function^4^.

Neither the current National Institute for Health and Care Excellence (NICE) guideline, nor the updated guideline from the American Academy of Paediatrics discuss any possible benefits of hyperbilirubinemia despite their comprehensive reviews of the literature^1,6^. The implication is that hyperbilirubinemia confers no benefit to the infant. Indeed, the NICE guideline makes specific mention of breastfeeding being a factor significantly associated with hyperbilirubinaemia, with no clear reason yet elucidated. It is clear that the potential benefit of neonatal jaundice requires further consideration and scientific exploration.

Neonates are prone to overwhelming bacterial sepsis in the first few days of life, so-called “early-onset” sepsis. The most common bacteria involved in early-onset sepsis remains *Streptococcus agalactiae*, commonly referred to as Group B *Streptococcus*^7^. It is well recognised that bacterial growth can be detrimentally affected by the presence of bile, and indeed bile resistance is a commonly used phenotypic tool in differentiating gastrointestinal organisms^8^. The mechanisms by which bile interferes with bacterial growth are multifactorial, but principally involve damage to the cell membrane, with the result being that Gram-negative organisms appear inherently more resistant to bile than Gram-positive organisms^8^.

We hypothesised that hyperbilirubinemia in the early neonatal period may confer an advantage to the host by negatively influencing bacterial replication and hence improving host survival from bacterial sepsis. We designed an *in-vitro* study to test this hypothesis utilising organisms derived from neonatal blood cultures.

## Methods

### Bacterial Isolates

Patient-distinct GBS strains were identified from septic neonates from Aberdeen Maternity Hospital, UK between January 2009 and March 2011. Isolates were retrieved from Medical Microbiology and classified as per their formal microbiology report identity, generated from VITEK®2 sensitivity analysis (BioMerieux). Since no clinical data was utilised in this study and all work was *in-vitro*, no ethics approval was required.

### Bilirubin Impact on Growth and GBS Transcriptome and Proteome Analysis

Bacterial isolates were grown on 5% horse blood, Columbia blood agar (CBA) plates, harvested, and re-suspended in nutrient broth (Oxoid) to achieve a bacterial concentration of ~10^3^ CFU/ml. 100 µl of each bacterial suspension was then plated in triplicate onto CBA plates as well as onto CBA plates which contained defined concentrations of bilirubin (Sigma-Aldrich) (0, 25, 50, 100, 200, 300, 400 µmol/l) dissolved in DMSO + 1 M NaOH. CBA plates with just solvent vehicle (DMSO + 1 M NaOH) were also included as controls. All bilirubin plates were prepared in a darkened environment and immediately covered with aluminium foil to prevent bilirubin degradation. After inoculation, the plates were again covered immediately and incubated aerobically in the dark at 37 °C overnight, upon which time the total number of colonies on each plate was recorded. Statistical analysis was performed using SPSS version 24. Following assessment within the plate model, the impact of bilirubin on GBS growth was then assessed within liquid culture. GBS strains were inoculated into liquid media containing fetal bovine serum supplemented with horse blood to 5% (v/v) with the addition of bilirubin to a final concentration of 100 μM dissolved in vehicle (DMSO and 1 M NaOH). A second liquid media (Solvent Control) containing the vehicle but no bilirubin was also used. Liquid culture was also used to assess the impact of bilirubin on GBS physiology. For both growth and GBS physiology studies, the GBS inoculation concentration was approximately 8 × 10^5^ CFU/mL (colonies harvested into nutrient broth, OD600 adjusted to 0.01, then broth added to media to a final volume of 10% v/v). These were incubated at 37 °C on a shaking plate (~140 rpm) for up to 24 hours. Experiments were performed in triplicate using 3 independent GBS isolates. The stability of bilirubin was determined in the liquid culture model over the study timeframe. Total bilirubin concentrations of (0, 25, 50, 75, 100, 200, 300, 400 µmol/l) were assessed in triplicate using the Diazo-bilirubin method^9^. All liquid culture experimental work was undertaken in foil wrapped tubes in dim light/dark conditions to minimise photo-oxidation of the bilirubin solution. Bilirubin solutions were also freshly prepared immediately prior to each experiment.

### RNA extraction and purification

After incubation, bacteria were harvested and total RNA was extracted, purified and on-column DNase treated using the QIAgen RNeasy mini prep kit according to the manufacturer’s instructions (Qiagen, Valencia, CA, USA). Three independent replicates for each GBS isolate were performed. The total RNA was then subjected to a second DNase I treatment with Turbo DNase (Applied Biosystems/Ambion) and were purified using the Qiagen RNeasy MinElute clean-up kit (Qiagen). RNAs were quantified using the NanoDrop ND-1000 spectrophotometer (Thermo Fisher Scientific, Waltham, MA, USA). RNA quality was evaluated on Tapestation 2200 with R6K screentape (Agilent Technologies Inc., Santa Clara, CA, USA), and all RNAs samples were deemed high quality (RIN ≥ 9).

### Bacterial mRNA Library Preparation and RNA-Seq

Ribosomal RNA (rRNA) was depleted using an Epicentre® Ribo-Zero™ rRNA Removal Kit (Bacteria) according to the manufacturer’s instructions (Illumina, San Diego, CA, USA). Briefly, samples in Ribo-Zero rRNA removal solution were incubated at 68 °C for 10 min followed by a 15 min incubation at room temperature. Samples were incubated with the prepared microsphere beads at room temperature for 10 min, followed by 50 °C for 10 min and column filtration. The final purification of eluted rRNA-depleted mRNA was performed by ethanol precipitation.

Strand-specific cDNA libraries were generated from rRNA-depleted mRNA samples using Ion Total RNA-seq Kit v2 for Ion Proton and Ion Xpress RNA-seq barcodes, following the manufacturer’s instructions (Ion Torrent, Thermo Fisher Scientific, Waltham, MA, USA). Barcoded libraries were quantified on the Tapestation 2200 with D1000 or HSD1000 screentapes (Agilent Technologies Inc., Santa Clara, CA, USA). Template preparation was performed on the Ion Chef and RNA-Seq was performed on the Ion Proton with Proton I chip and 200-bp single end reads by the Centre for Genome Enabled Biology and Medicine at the University of Aberdeen (Aberdeen, UK). Base calling was performed and fastq files generated using Torrent Suite v 4.2.1.

Read mapping was performed on Galaxy at the high-performance research computing centre at the University of Aberdeen. Trim Galore! version 0.4.0^10^ was used to trim sequences with a quality score < 20, and sequences <20 bp after trimming were filtered. TopHat2 version 2.0.10^11^, with minimum and maximum intron size of 20 and 120, respectively, was used to align the filtered reads to the *S*. *agalactiae* 2603 V/R reference genome with annotation in gff3 format (Ensembl bacteria build 25^12^; ftp://ftp.ensemblgenomes.org/pub/bacteria/release-25/). Feature counts were performed using HTSeq version 0.6.1p1^13^ with a minimum alignment quality of 20 and reads counted if they spanned a particular gene in whole or in part, or if the read spanned a gene exon-exon junction (intersection non-empty overlap mode).

### Statistical Analysis of RNA-Seq Data

Differential expression was calculated using edgeR version 3.6.8^14^, built under R version 3.1.1 and a GLM model with two factors: strain and treatment. Normalisation of read counts based on library size was performed within edgeR using TMM method. Dispersion and model fit were calculated by edgeR using robust = TRUE^15^. Gene Ontology enrichment, using Fisher’s exact test, was performed with Partek Genome Suite version 6 (Partek Inc, USA) using Gene Ontology annotation for *S*. *agalactiae* downloaded from www.ebi.ac.uk/GOA. Benjamini and Hochberg false discovery rate^16^ was used to correct for multiple testing (FDR ≤ 0.05).

### Proteomics

#### Preparation of samples for protein extraction

Whole cell protein extracts were prepared from three independent cultures of each GBS isolate. The bacterial cells were pelleted from the culture media by centrifugation at 2500 × g for 10 min and washed with sterile water, to lyse blood cells, then with sterile PBS. Preparation of protein extracts and analysis by 2D gel electrophoresis was undertaken within the University of Aberdeen Proteomics facility following protocols described previously^17^.

#### Functional/pathways analysis

To gain further insight into the effects of bilirubin on GBS, we performed pathway enrichment analysis using Partek Pathway in Partek Genomic Suite version 6 using the KEGG database and *S*. *agalactiae* gene annotation^18^.

## Results

**Figure 1:**
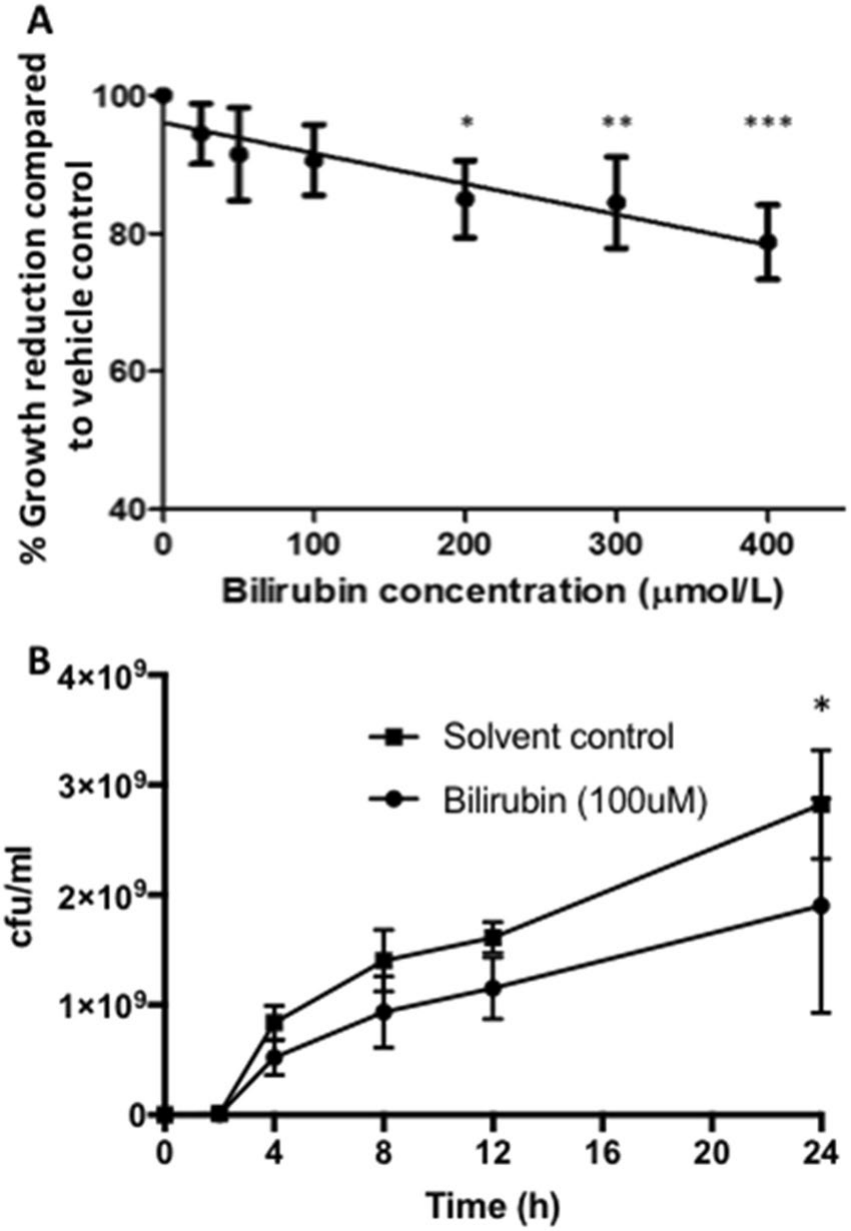
Effect of bilirubin on growth of GBS. (**A**) Solid media model. Total number of colonies were determined following overnight incubation of blood agar plates containing bilirubin at concentrations ranging from 0–400 µmol/l bilirubin dissolved in DMSO/1 M NaOH. Results are presented as percentage change in growth from 100% on DMSO/1 M NaOH control. N = 7 isolates with experiments repeated in triplicate. Values are presented as mean+/− SEM. All values depicted with an asterisk were statically significant (independent sample T-test). * = 0.011, ** = 0.024 and *** = <0.001. (**B**) Liquid culture model. Total number of colonies were determined following incubation for up to 24 hrs of Blood/serum containing bilirubin at a concentration of 100 µmol/l bilirubin dissolved in DMSO/1 M NaOH or Blood/serum containing vehicle (DMSO/1 M NaOH). Results are presented as colony forming units (CFU). N = 3 isolates with experiments repeated in triplicate. Values are presented as mean + /− SEM. All values depicted with an asterisk were statically significant (independent sample T-test). * = 0.02.

Seven GBS isolates were obtained from the Medical Microbiology laboratory at Aberdeen Royal Infirmary from neonatal sepsis cases between January 2009 and March 2011. These isolates were assessed for bacterial growth in the presence of varying concentrations of bilirubin (0–400 µmol/l) using a plate-based model to span the clinical range seen in the context of neonatal hyperbilirubinaemia. Bilirubin caused a significant reduction in GBS growth at concentrations above 100 µmol/l when compared to growth in the absence of bilirubin (Fig. 1A). In order to assess the impact of bilirubin on GBS growth in a more clinically reflective model of EOS, we devised a novel liquid culture system to more closely simulate hyperbilirubinaemic blood. We found that the highest concentration of bilirubin that achieved acceptable stability over the duration of the experiment (24 hrs) was 100 µmol/l (Supplementary Fig. 1). Importantly, recent evidence suggests a switch from a potent antioxidant to a pro-oxidant function for bilirubin at concentrations around 100 µmol/l^5^. We therefore assessed the effects of 100 µmoll/l bilirubin on GBS growth in the liquid-culture model. As with the plate-based model, bilirubin caused a significant reduction in GBS growth, compared to vehicle alone. At 24hrs there was a 33% reduction in GBS growth (p = 0.02; Fig. 1B).

In order to elucidate the impact of bilirubin on GBS physiology, we performed a whole bacterial transcriptome and proteome analysis of GBS isolates grown in the presence or absence of bilirubin. We undertook this analysis in combination with gene network pathway analysis to gain a comprehensive picture of the underlying molecular mechanisms involved in the response of GBS to bilirubin exposure. We elected to perform these analyses on three GBS isolates which were chosen at random from the collection, as there were minimal differences in the growth profile between the seven strains based on the plate-model. We performed a comprehensive assessment of the effects of 100 µmoll/l bilirubin on the transcriptome and proteome of GBS.

Analysis of gene expression by RNA-seq identified 19 genes that were differentially expressed at 100 µmol/l bilirubin concentration when compared with vehicle control (FDR adjusted p ≤ 0.1) (Table 1). The majority of genes (18/19) had increased expression levels in the presence of bilirubin. Most of the over-expressed genes were involved in hydrolase and transport functions. One of the most significant differentially expressed genes was over-expression of *sag*1901 a glucuronyl hydrolase (unsaturated chondroitin disaccharide hydrolase/unsaturated glucuronyl hydrolase (UGL; GH88 family)), which is involved in degrading glycosaminoglycans and sulphated substrates. Previous investigations of chitosan, a natural polysaccharide with structural characteristics similar to glycosaminoglycans, have shown that it can effectively bind bilirubin in *in-vitro* models^19^. It is therefore possible that, within our liquid model system, GBS is increasing glucuronyl hydrolase production in response to the presence of bilirubin as an innate defence mechanism in order to bind the perceived toxin.

**Table 1:** *S*. *agalactiae* genes differentially expressed in response to bilirubin.

ID	Name	log fold change	p-value	FDR
SAG1901	Glucuronyl hydrolase	1.13	1.29E-07	1.29E-04
SAG1807	Hypothetical protein	1.30	1.35E-07	1.29E-04
SAG1898	PTS system transporter subunit IID	1.87	1.06E-06	5.81E-04
SAG0547	Hypothetical protein	−1.32	1.22E-06	5.81E-04
SAG0118	Ribokinase	1.12	2.96E-05	1.13E-02
SAG1902	PTS system transporter subunit IIA	1.34	4.07E-05	1.29E-02
SAG1899	PTS system transporter subunit IIC	1.83	5.82E-05	1.59E-02
SAG0169	Formate acetyltransferase	1.32	1.26E-04	2.46E-02
SAG1350	Surface antigen-like protein	2.02	1.28E-04	2.46E-02
SAG0680	Hypothetical protein	0.41	1.29E-04	2.46E-02
SAG0325	Pyruvate formate-lyase-activating enzyme	1.81	1.91E-04	3.31E-02
SAG1900	PTS system transporter subunit IIB	1.55	2.67E-04	4.02E-02
SAG0795	Hypothetical protein	2.83	2.73E-04	4.02E-02
SAG1925	Sugar ABC transporter ATP-binding protein	2.02	3.37E-04	4.60E-02
SAG1441	Maltose/maltodextrin ABC transporter maltose/maltodextrin-binding protein	2.19	4.69E-04	5.91E-02
SAG1690	PTS system transporter subunit IIABC	2.01	5.22E-04	5.91E-02
SAG2072	Uridine phosphorylase	1.70	5.26E-04	5.91E-02
SAG0331	Formate acetyltransferase	1.65	8.21E-04	8.43E-02
SAG2014	Hypothetical protein	1.17	8.39E-04	8.43E-02

Genes encoding transport proteins were also upregulated in the presence of bilirubin. These included *sag*1925 and *sag*1441, which both encode components of ABC transporters involved in the transport of sugars. ABC transporters are known to translocate a wide variety of endogenous and exogenous substrates across cell membranes and have previously been linked to protecting cells from bilirubin toxicity^20^. Up-regulation in the presence of bilirubin could reflect expulsion of toxic bilirubin from bacterial cells, or an increased requirement for sugar uptake from the culture medium to compensate for increased metabolic demands.

Furthermore, the transcription of genes encoding carbohydrate transport proteins (*sag*1898, *sag*1902, *sag*1899, *sag*1900, *sag*1441 and *sag*1690) were also found to be significantly upregulated. These genes are the five subunits and components of the phosphotransferase system: PTS transporter subunits IIA – IID, and IIABC components. The PTS transport system is widely spread among bacteria of many different species, and it can be used to transport multiple types of carbohydrates into the cell.

### Proteomic assessment of bilirubin impact

**Figure 2:**
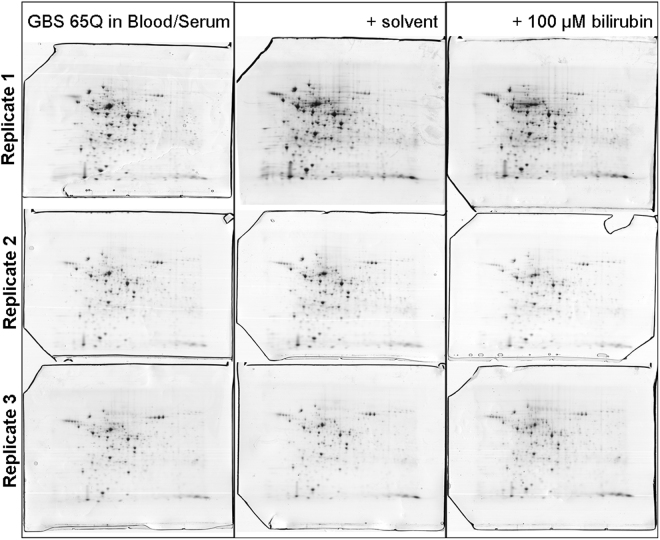
Representative 2D Gel electrophoresis image from single isolate (65Q) incubated in three types of media, in triplicate. Samples were incubated for 24 hours at 37 °C in blood serum (media), solvent control (DMSO/1 M NaOH) (solvent) or 100 µmol/l bilirubin dissolved in DMSO/1 M NaOH. Total protein from each sample was assessed for quality by 1DGE prior to resolution by 2DGE and staining by Coomassie Brilliant Blue. These images were used for comparison of protein profiles with and without exposure to bilirubin by Progenesis SameSpots.

**Figure 3:**
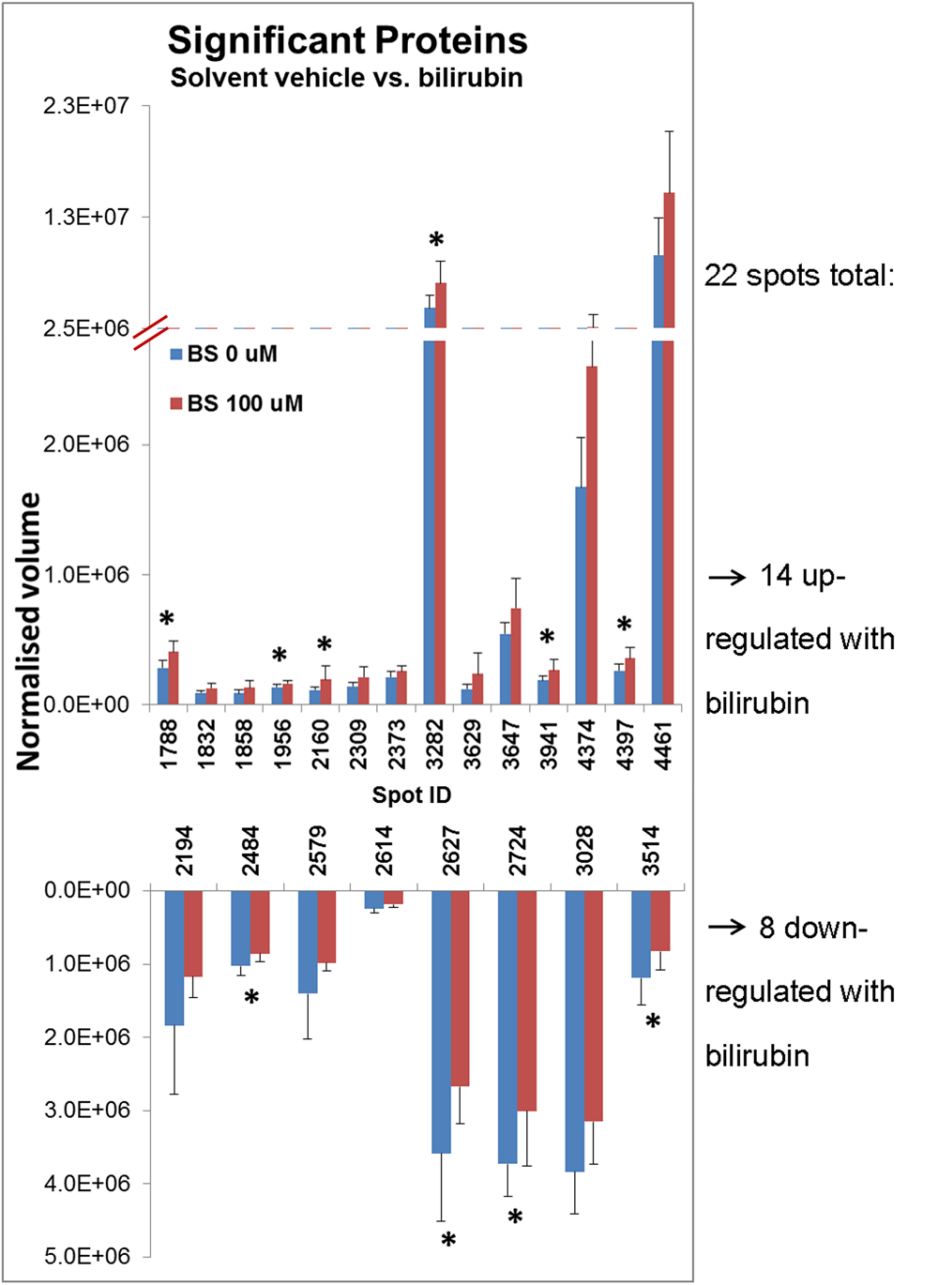
Average normalised spot volumes of GBS 19 Y, 63 L and 65 Q, isolates each in triplicate ± standard deviation. ANOVA analysis identified 22 significant spots, 14 were up-regulated in the presence of bilirubin, and 8 were down-regulated.

Changes in the GBS proteome of the clinical isolates following bilirubin exposure were evaluated. The total cellular fractions of a representative GBS isolate are depicted in Fig. 2. Alteration in the abundance of 22 proteins was observed when GBS was exposed to bilirubin when compared to solvent control. Fourteen proteins were increased, while 8 were decreased in the presence of bilirubin (Fig. 3; Table 2). A subset of 10 proteins that were significantly altered in the presence of bilirubin, with significance p < 0.025, were further analysed by MALDI-TOF/TOF MS. In this subset, half were upregulated and their identities are given in Table 3. Several of the proteins up-regulated in the presence of bilirubin were related to transporters, validating the gene expression changes described previously. Peptide ABC transporter ATP-binding, ABC transporter permease and Iron ABC transporter ATP-binding protein. Other up-regulated proteins included the enzymes phosphoglycerate kinase and s-ribosylhomocysteinase, and also the regulators and molecular chaperones GntR family transcriptional regulator and molecular chaperone GroEL (Table 3). Proteins which were down-regulated in the presence of bilirubin were related to regulation, synthesis or enzymatic activities. These included single-stranded DNA binding protein, N-acetyl neuramic acid synthetase NeuB, and transketolase, ornithine carbamoyltransferase, and dTDP-4-dehydrorhamnose reductase. The functional importance of these alterations in the proteome in response to bilirubin was further explored by gene ontology and pathways analyses.

**Table 2:** *S*. *agalactiae* proteins altered in response to bilirubin.

Spot #	Fold-change	p-value
Significantly higher expression in the presence of bilirubin		
1788	1.545	0.002
3282	1.709	0.012
3941	1.403	0.015
2160	1.704	0.02
4397	1.369	0.021
1832	2.67	0.027
2309	1.493	0.032
3647	1.51	0.034
3629	1.944	0.037
1858	2.769	0.038
4461	1.62	0.046
2373	1.207	0.048
**Significantly lower expression in the presence of bilirubin**		
2484	1.223	0.018
3514	1.444	0.023
2724	1.239	0.024
2627	1.341	0.024
3028	1.217	0.032
2194	1.792	0.039
2614	1.316	0.043
2579	1.428	0.047

**Table 3:** LC-MS identified proteins altered in response to bilirubin.

Spot #	Protein identity (RefSeq protein ID, RefSeq gene ID)	MUDPIT score	Sequence coverage (%)	Fold-change	p-value
**Significantly higher expression in the presence of bilirubin**					
1788	ABC transporter permease (WP_000897811, no RefSeq gene ID)	37	5	1.545	0.002
3282	Phosphoglycerate kinase (AAL85687, upp)	79	5	1.709	0.012
3941	S-ribosylhomocysteinase (WP_000159883, luxS)	186	39	1.403	0.015
2160	Molecular chaperone GroEL (WP_002435951, groEL)	105	9	1.704	0.021
4397	GntR family transcriptional regulator (WP_000312253, DX05_05330)	48	29	1.369	0.025
**Significantly lower expression in the presence of bilirubin**					
2484	N-acetyl neuramic acid synthetase NeuB (WP_000262516, neuB)	326	55	1.223	0.018
3514	Single-stranded DNA-binding protein (WP_000609585, ssb1)	194	73	1.444	0.023
2724	dTDP-4-dehydrorhamnose reductase (WP_000600895, rfbD)	244	46	1.239	0.024
2627	Ornithine carbamoyltransferase (WP_000793622, arcB1)	193	42	1.341	0.024
3028	Iron ABC transporter ATP-binding protein (WP_000114500, no RefSeq gene ID)	151	35	1.217	0.032

10 significant proteins from across three GBS isolates were selected and identified by LC-MS and MUDPIT database.

**Figure 4:**
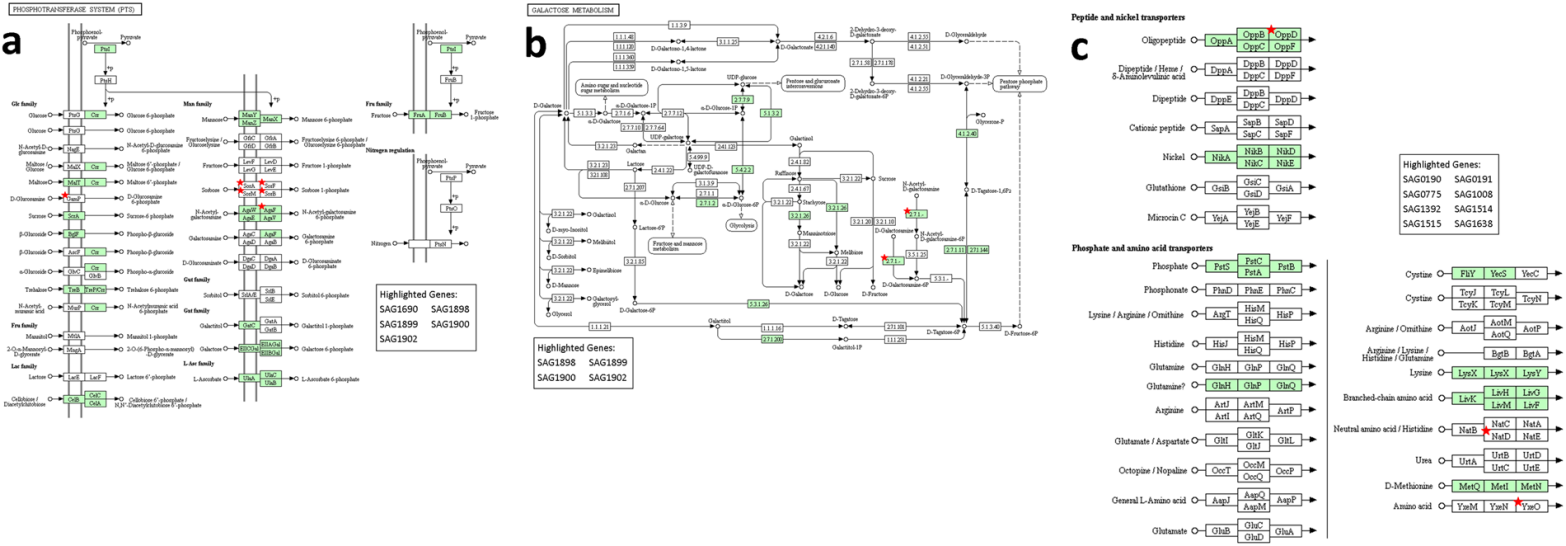
KEGG pathways analysis (**A**) Phosphotransferase system, (**B**) Galactose Metabolism, (**C**) Selected ABC transporters (KEGG is described in the following paper: Kanehisa, M. & Goto, S. KEGG: Kyoto encyclopedia of genes and genomes. *Nucleic Acids Res*. **28**, 27–30 (2000).

Gene ontology analysis was performed on the set of differentially expressed genes obtained from the RNA-seq analysis, combined with the genes encoding the proteins identified as differentially expressed in response to bilirubin. This combined transcriptome and proteome analysis identified carbohydrate metabolism and transport, including the phosphotransferase system (PTS), as significantly altered in response to bilirubin (Table 4). Additionally, nucleotide metabolic processes, essential for cell growth and replication, were significantly altered. Interestingly, the biological process involving catabolism of arginine (a precursor of nitric oxide) to ornithine, a precursor of polyamines, was significantly altered in response to bilirubin (Table 4). Pathway enrichment analysis of *S*. *agalactiae* KEGG pathways^18^ identified galactose metabolism, the PTS system and ABC transporters as significantly altered in GBS in response to bilirubin (Fig. 4, Supplementary Table 1). These analyses further suggest carbohydrate metabolism and transport as key factors in the response of the pathogen *S*. *agalactiae* to bilirubin.

**Table 4:** GO enrichment analysis depicting enriched biological processes and molecular functions which are altered in response to bilirubin exposure.

GO Category	Enrichment p-value
**Biological Processes**	
PTS system	2.42E-04
Carbohydrate phosphorylation	4.97E-02
UMP salvage	4.97E-02
Nucleotide catabolic process	4.97E-02
Arginine catabolic process to ornithine	4.97E-02
Carbohydrate transmembrane transport	5.13E-02
Guanosine tetraphosphate metabolic process	6.57E-02
**Molecular Functions**	
Catalytic activity	1.40E-03
Protein-N(PI)-phosphohistidine-sugar phosphotransferase activity	4.30E-02
Carboxyl- or carbamoyltransferase activity	4.97E-02
Transferase activity, transferring pentosyl groups	6.57E-02
Single-stranded DNA binding	9.70E-02

GO categories enriched for the set of 19 genes differentially expressed and 12 genes corresponding to proteins differentially expressed.

## Discussion

We have shown in an *in-vitro* study utilising neonatally-derived blood culture isolates that bilirubin may have antibacterial properties against GBS. We then used a combined transcriptomic/proteomic approach to characterise the functional impact of bilirubin exposure on GBS physiology by developing a novel *in-vitro* culture model of hyperbilirubinaemia. In doing so, we have identified particular adaptive responses within GBS, highlighting in particular gylycosaminoglycan degradation, ABC transporters, and carbohydrate metabolism and transport. These suggest putative mechanisms by which GBS adapts to bilirubin exposure, and offer potential therapeutic targets for exploitation in future models of hyperbilirubinaemia and sepsis.

The liquid model has two main benefits over the plate-based model. Firstly, the bacteria and the bilirubin are in free suspension which is more reflective of blood. Secondly, the addition of serum adds albumin, an important binding agent for bilirubin, more closely reflecting the situation *in-vivo*. The level of albumin in this model was not measured or controlled however, and is something that warrants consideration in future studies, particularly with a view to approaching those seen in neonates with sepsis. Albumin is known to bind bilirubin *in-vivo*, however albumin levels can vary dramatically in neonates depending on gestational age, nutritional state and other physiological parameters.

This study was principally limited by being entirely *in-vitro*, and on a small number of GBS isolates, nevertheless it demonstrates important proof of concept data supporting the hypothesis that bilirubin may have an under-recognised antibacterial role of potential importance in neonatal sepsis. Further validation of the effects of hyperbilirubinaemia on GBS growth should now be assessed on a larger collection of GBS strains. A further limitation is the lack of information relating to the actual GBS strains used within the study. The strains were clinically-derived early-onset sepsis isolates. We accept that further work on GBS at a strain level might prove interesting, however this was deliberately a clinically-orientated preliminary study, so used information derived and used routinely in clinical practice. The information provided on routine blood culture reports in the healthcare setting, from which these isolates were derived, includes species identity and antibiotic sensitivity profiles and does not go to strain level for GBS. This was therefore the level of bacterial information used a baseline in this study. Further work is now required to explore this concept in animal models of combined hyperbilirubinaemia and sepsis, with a broader range of GBS strains, and with more species of Gram-positive and Gram-negative pathogens, before clinical studies can be undertaken. Such clinical studies might feasibly randomise neonates at high risk of developing sepsis to high or low tolerance thresholds of hyperbilirubinaemia to look at their subsequent rates of sepsis and the resultant course of illness.

Although GBS is responsible for a significant number of early-onset sepsis cases, it is not the only pathogen linked with this. *Escherichia coli* accounts for almost a quarter of early-onset sepsis episodes and is the most common cause of mortality in this condition^21,22^. We had previously reported that bilirubin had no effect on bacterial growth in Gram-negative *E*. *coli* clinical strains when compared to GBS strains^23^. Other studies indicate that bilirubin can actually act as a protectant to *E*. *coli* strains whilst being highly toxic towards Gram-positive bacteria including *Enterococcus faecalis*^24^. The proposed mechanism of protection of *E*. *coli* was through neutralisation of host ROS production, whilst the toxic impact on the Gram-positive organisms was thought to be caused by the lipophilic properties of bilirubin. This highlights the pleotropic effects that small molecules metabolites such as bilirubin can have on different bacterial species and reinforces the need to investigate different bacterial groups rather than relying on extrapolating previous findings.

Hyperbilirubinemia is a recognised feature of sepsis in neonatal, paediatric and adult populations and indeed there is evidence to suggest that haem catabolism is increased in the critically ill with a resultant rise in serum bilirubin^25^. It may be that hyperbilirubinemia in this context reflects an innate immune response rather than an epiphenomenon, a concept that warrants further exploration, however we should also remain mindful that the level of hyperbilirubinaemia appears itself to be an independent prognostic indicator in severe sepsis^26^. Other investigators have explored the role of bilirubin in endotoxic shock. Lanone *et al*.^27^ utilised homozygous jaundice Gunn rats (which display elevated plasma bilirubin levels of ~150 µmol/l because of deficient glucuronyl transferase activity) in addition to Sprague-Dawley rats subjected to exogenous bilirubin administration (5 mg/kg over 1 minute then 50 mg/kg/hr over 3 hours), achieving ~80 µmol/l serum bilirubin, to demonstrate that when subjected to *E*. *coli* lipopolysaccharide (LPS), there was a significant decrease in LPS-stimulated nitric oxide (NO) production. This effect was due to an attenuation of inducible NO synthase (NOS2) by the effect of bilirubin on NADPH oxidase, which is itself involved in NOS2 induction. The same effect was also witnessed *in-vitro*. Physiologically, the reduction in NO production was apparent in a significantly higher blood pressure in both hyperbilirubinaemic rat groups after LPS administration. The possibility of utilising bilirubin as a treatment for endotoxic shock was explored by Kadl *et al*.^28^ who challenged Balb/C mice with a sub-lethal dose of *E*. *coli* LPS and either treatment with 40 mg/kg bilirubin or a control infusion. This study showed multiple clinical improvements in the bilirubin treated group where bilirubin seemingly attenuated the endotoxin-induced tissue injury and exerted potent anti-oxidant activity through a reduction in the expression of pro-inflammatory genes including IL-1β and TNF-α. It may be that induction of hyperbilirubinemia (or in the case of neonatology, tolerance of physiological hyperbilirubinemia) could be a valuable therapeutic tool in the treatment of sepsis, affecting the proliferation of Gram-positive organisms such as GBS, and with additional benefits in endotoxic shock due to Gram-negative sepsis. Furthermore, there is a growing body of evidence linking hyperbilirubinemia with several other physiological effects related to cellular pathways and homeostasis^29^. These include not only antioxidant effects but also anti-inflammatory, antiproliferative and immunomodulatory activities. Further exploration of the potentially wide-ranging positive role of bilirubin in host physiology is overdue and worthy of targeted study.

In an era where the phototherapy units used to treat neonatal jaundice are predominantly driven by light emitting diodes, the complications of hyperthermia and dehydration of the past are largely irrelevant. Use of phototherapy may therefore tend towards lower treatment thresholds to prevent the complications of hyperbilirubinemia, particularly where no “benefit” from hyperbilirubinaemia is perceived. Further studies are therefore needed to investigate whether hyperbilirubinemia may have potential benefits in the context of sepsis and, in view of our results, with particular reference to the development and progression of early-onset sepsis and, specifically, GBS sepsis in the neonate.

## Conclusion

Physiological hyperbilirubinemia may have beneficial effects in reducing the growth of pathogenic Group B *Streptococci*, whilst other evidence suggests that hyperbilirubinemia may be protective in Gram-negative endotoxic shock. The role that hyperbilirubinemia plays in protecting infants against sepsis requires further research.

## Electronic supplementary material

Supplementary Information
