# Supplementary material for: Adaptive response of neonatal sepsis-derived Group B Streptococcus to bilirubin
Source: Sci Rep. 2018 Apr 24;8:6470. doi: 10.1038/s41598-018-24811-3 (PMC5915570; doi:10.1038/s41598-018-24811-3)

**Adaptive response of neonatal sepsis-derived Group B Streptococcus to bilirubin**

Richard Hansen, Sophie Gibson, Eduardo De paiva Alves, Mark Goddard, Andrew MacLaren, Anne Marie Karcher, Susan Berry, Elaina S R Collie-Duguid, Emad El-Omar, Mike Munro and Georgina L. Hold

**Supplementary Table 1: Biological pathways altered in response to bilirubin**

| **Pathway Name** | **p-value** |
| --- | --- |
| Galactose metabolism | 4.56E-06 |
| Phosphotransferase system (PTS) | 5.22E-04 |
| ABC transporters | 1.28E-01 |

KEGG pathways^18^ enriched for the set of 19 genes differentially expressed and 12 genes corresponding to proteins differentially expressed.

**Supplementary Figure 1:** Assessment of stability of bilirubin over 24 hours – Bilirubin was dissolved in DMSO ± NaOH at varying concentrations (0 – 400 µM) in fetal bovine serum supplemented with defibrinated horse blood (5 % v/v total volume), n = 3 ± SEM. All liquid culture experimental work was undertaken in foil wrapped tubes in dim light conditions, just sufficient to allow safe working, to minimise photo-oxidation of the bilirubin solution. Bilirubin solutions were also freshly prepared immediately prior to each experiment.


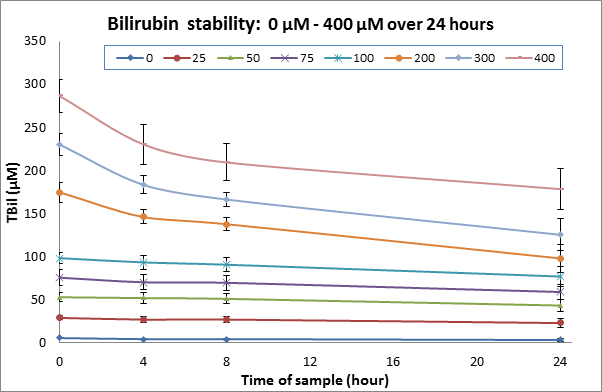


**Supplementary Figure 2:** 2D Gel electrophoresis image from remaining two isolates (19Y and 63L) incubated in three types of media, in triplicate. Samples were incubated for 24 hours at 37 °C in blood serum (media), solvent control (DMSO/1M NaOH) (solvent) or 100 µmol/L bilirubin dissolved in DMSO/1M NaOH. Total protein from each sample was assessed for quality by 1DGE prior to resolution by 2DGE and staining by Coomassie Brilliant Blue. These images were used for comparison of protein profiles with and without exposure to bilirubin by Progenesis SameSpots.


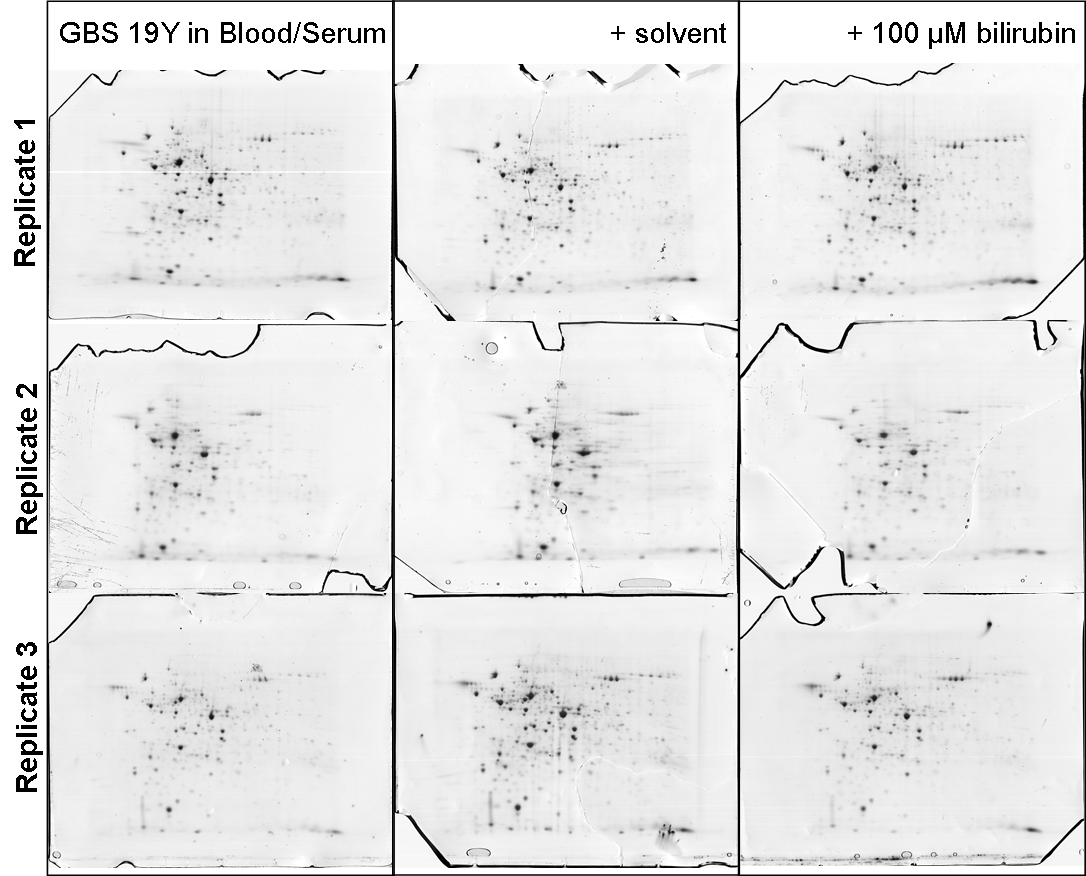


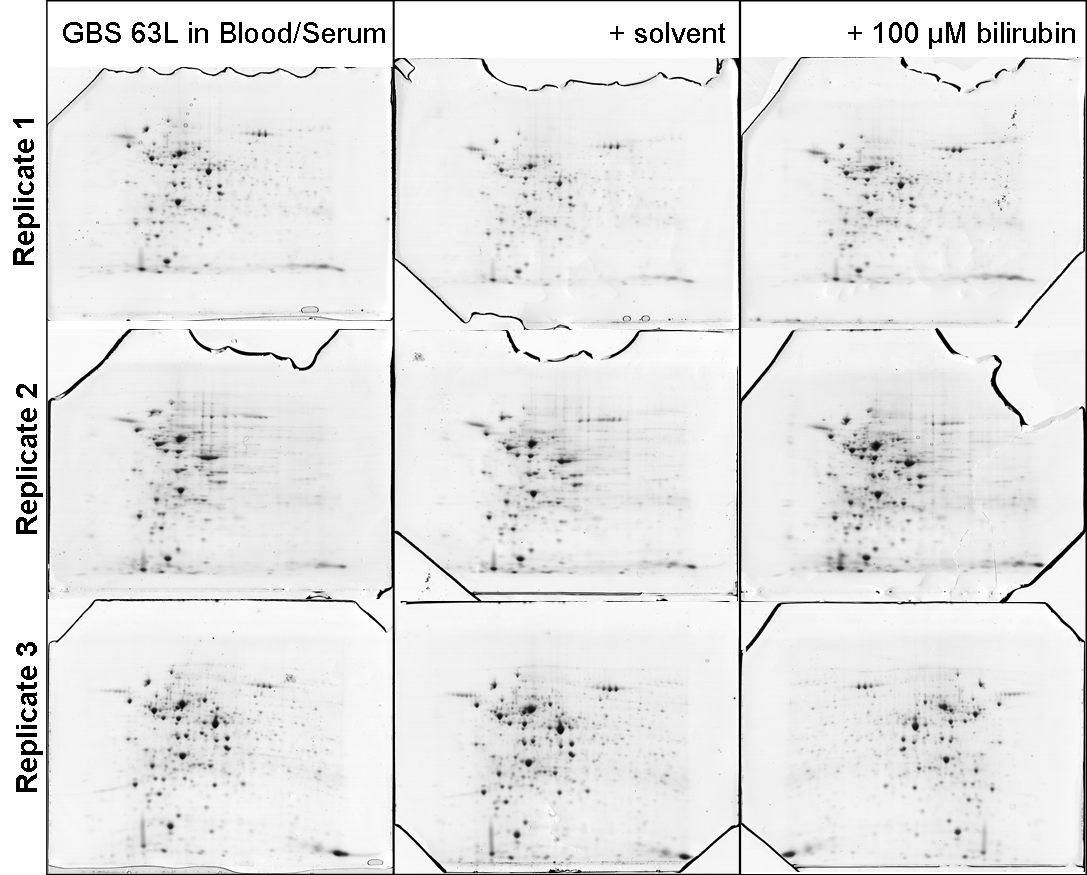

Supplement: Supplementary file 1 — Supplementary Information [file 41598_2018_24811_MOESM1_ESM.docx]
